# Parental attitudes to neoanus dilatations post-reconstruction in anorectal malformations (PANDA) study

**DOI:** 10.1007/s00383-025-06190-9

**Published:** 2025-09-17

**Authors:** H. Thakkar, V. Haffenden, S. Yardley

**Affiliations:** 1 Paediatric General Surgery, Evelina London Children’s Hospital, Guys & St. Thomas’ NHS Foundation Trust, London, United Kingdom; 2 Marie Curie Palliative Care Research Department, University College London, London, United Kingdom

**Keywords:** Anorectal malformations, dilatations, parents, caregivers

## Abstract

**Purpose:**

Some surgeons train parents to undertake routine neoanus dilatations following anorectal malformation surgery. Recent studies suggest this may not significantly reduce the incidence of strictures. This study sought to understand parental experiences of and perceptions about this intervention to guide best practise and inform further studies of clinical impact.

**Methods:**

This qualitative study, using reflexive thematic analysis was carried out in a single institution. Five parents took part in an audio-recorded focus group with 13 families subsequently taking part in audio-recorded semi-structured interviews. Concurrent analysis of verbatim transcripts guided iterations in data generation.

**Results:**

Four major themes were identified; (1) parents understood the importance of dilatations; (2) parents experienced distress with anxiety, fear of causing harm and guilt; (3) parents perceived a traumatic reaction from infants including anticipatory stress; and (4) some parents perceived detrimental impact on inter-parental relationships with an imbalance in responsibilities for dilatations.

**Conclusions:**

Parents who accept the importance of dilatations, are placing trust in their clinicians’ guidance despite their negative experiences of the intervention and perceived relational costs between themselves and their child. As the quantitative clinical evidence for neoanus dilatation remains, at best, equivocal, we do not recommend routine dilatations post-reconstructive surgery for anorectal malformations.

## Introduction

In 1982, deVries and Pena first described the posterior sagittal anorectoplasty (PSARP) approach when reconstructing anorectal malformations [1]. As part of the reconstruction, routine post-operative dilatations were also recommended to reduce the risk of stricture formation and gradual dilatation of the anoplasty without damaging the surrounding muscle. The PSARP approach has since been popularised across the globe [2]. A 2015 survey conducted across 16 European countries revealed 88% routinely recommending post-operative dilatations [3] with similar numbers also prescribing this practise in a 2016 survey of European surgeons [4].

A case–control study between two UK centres with differential practise on routine dilatations reported no significant difference in the rate of stricture formation [5]. Up to a 1/3 of patients in each centre required unplanned dilatations under GA with just over a 1/5 needing further surgery irrespective of whether patients were reported to have received routine dilatations versus only for specific indications.

In 2021, Ahmad et al. [6] conducted a randomised-controlled trial to determine if routine post-operative dilatations were necessary to reduce the rate of stricture formation. The authors reported a similar stricture rate in both treatment arms and concluded that routine dilatations were not necessary.

Whereas attempts have been made by paediatric colorectal surgeons to objectively determine the need for dilatations, there is a paucity of data reporting the subjective impact on the patient and parents who perform the dilatations. In 1994, Diseth et al. conducted a psychosocial study in patients who had undergone surgery for a low anorectal malformation [7]. In those undergoing dilatations, parental experiences were largely negative with mothers in particular finding the procedure difficult to perform due to the perception of evoking pain in their child. Several mothers also explained the “great protestations” they witnessed from their child. The same group of authors conducted a further study in 17 adolescents who had undergone dilatations following reconstructive surgery [8]. Parents again negatively described their emotions towards performing dilatations with a physical struggle often reported during the procedure. Parents felt the dilatations negatively impacted their relationship with their child and also adversely affected their inter-parental relationship.

With emerging studies now reporting the equivocal impact of dilatations in reducing the rate of stricture formation [5, 6], we sought to understand parental experiences of and perceptions about this intervention to guide “patient” (parent/child) personalised care and clinical best practise.

## Methods

A qualitative study design incorporating reflexive thematic analysis was implemented after attaining Health Research Authority (HRA) ethical approval (23/YH/0169).

### Participants

Participants were identified through a prospectively maintained, departmental database. Inclusion criteria were any patients who had undergone reconstructive surgery for an anorectal malformation at our institution with parents being asked to routinely perform dilatations at home. Any patients directly under the care of the research team were excluded from participation to avoid conflicts of interest. Any patient who had undergone a reconstruction > 5 years prior to the study commencement data in 2024 were also excluded to reduce recall bias. Although arguably parental memories of events hold greater meaning in relation to social impact than ‘factual’ records of event, it remained important for participants to be able to recall details of their experiences. Eligible families were contacted for participation in the study via letters and a follow-up telephone or email. After attaining informed consent, an initial focus group was conducted.

### Data collection

A topic guide was created for the focus group with two moderators VH [female] and HT [male] conducting it virtually online through Microsoft Teams (Microsoft). The focus group was conducted online to allow as many families to participate saving both time and travel costs associated with the in-person setting. The discussion was audio-recorded and an automated transcript generated for analysis. This was cross checked by HT for accuracy and edited to produce a verbatim final version. Table 1 summarises the questions used as part of the focus group.

**Table 1:** Summary of topic guide for the focus group

***Informing/teaching—10 min***
Could you tell me about how you found out you would need to perform dilatations; were you asked by your surgeon? What did you understand to be the reason for performing dilatations? Who informed you of this; surgeon, clinical nurse specialist (CNS)? Could you describe to me how you learnt to perform the dilatations?
***Performing dilatations—20 min***
How did you feel when performing the dilatations? How did you interpret your role in performing the dilatations? Were there any aspects of the procedure you found especially easy or difficult? How did it fit in with your home set up and did you do this alone or have help from a partner/family member/carer? How did your baby respond to the dilatations? Did you have any concerns about performing the dilatations? Were there any times you felt unable to do them? Why? Did you seek any support in performing the dilatations? If so where from and was this beneficial?
***Future—10 min***
Has performing the dilatations changed anything about your child’s behaviour now; either socially/emotionally or around toileting? Has performing the dilatations had any positive or negative influence on your relationship with your child? Has performing dilatations had any positive or negative influence on other relationships with partner/family?

The results of this focus group were analysed to devise a parent orientated topic guide, sensitive to their concerns and perspectives, to be used to conduct a one-to-one, virtual, semi-structured interview by HT (Table 2).

**Table 2:** Summary of topic guide for the interviews

How did you first feel when you found out about your child having an ARM?
When did the surgeon first mention about the need for dilatations? Do you remember this well and if so what were your thoughts?
Did you receive any more explanations either before or after the reconstruction about performing this? Did the team discuss the aims and purpose of the dilatations?
Were you shown how to perform them and given any support?
Were you able to perform the dilatations and if so how did you find that?
Did you feel it was having any impact on your child?
Was there any impact on your family?
How did performing dilatations fit in with the rest of your life—other children and work?
Did you seek any additional support to perform the dilatations?

Was there any impact on your relationship with your partner? (added after first interview)

### Researcher characteristics

VH is a surgical research fellow who completed a qualitative research methodology course as part of this research study. HT is a Consultant Neonatal and Paediatric Surgeon who has completed online training to conduct focus groups and interviews as part of qualitative research. HT is the lead colorectal surgeon within the department and does not routinely performing dilatations on his patient cohort. SY is a Clinical Academic with extensive experience and expertise in conducting qualitative research, particularly using sociocultural theories to understand gaps between expectations and experience in healthcare.

### Data analysis

The focus group was conducted in June 2024 and lasted for 1 h and 14 min. The interviews were conducted between September 2024 and January 2025. The mean duration of the interviews was 18 min (7.5–41 min). Concurrent analysis of verbatim transcripts guided iterations in data generation. Reflexivity guided initial en vivo coding. The transcript of the focus group was analysed by HT and VH in detail following familiarisation of it by all authors. Research team meetings were held to reach consensus on the interview topic guide as a product of this analysis. For the interviews, Data familiarisation was undertaken by HT and SY and subsequently systematically coded line-by-line by HT. Initial themes were generated, discussed in research team meetings and these were then developed and reviewed further by HT. On reaching theoretical data saturation further reflexive thematic analysis, and cross comparison of the whole data set was undertaken utilising the NVivo (Lumivero) software package. Refining, defining and naming of themes was conducted by HT with the support of SY.

## Results

### Participants

Five parents participated in the focus group and 13 parents were subsequently interviewed. Four parents who were interviewed had also participated in the focus group. Four eligible families refused participation in the study with no specific reasons identified. No participants dropped out of the study.

### Themes

**Fig. 1:**
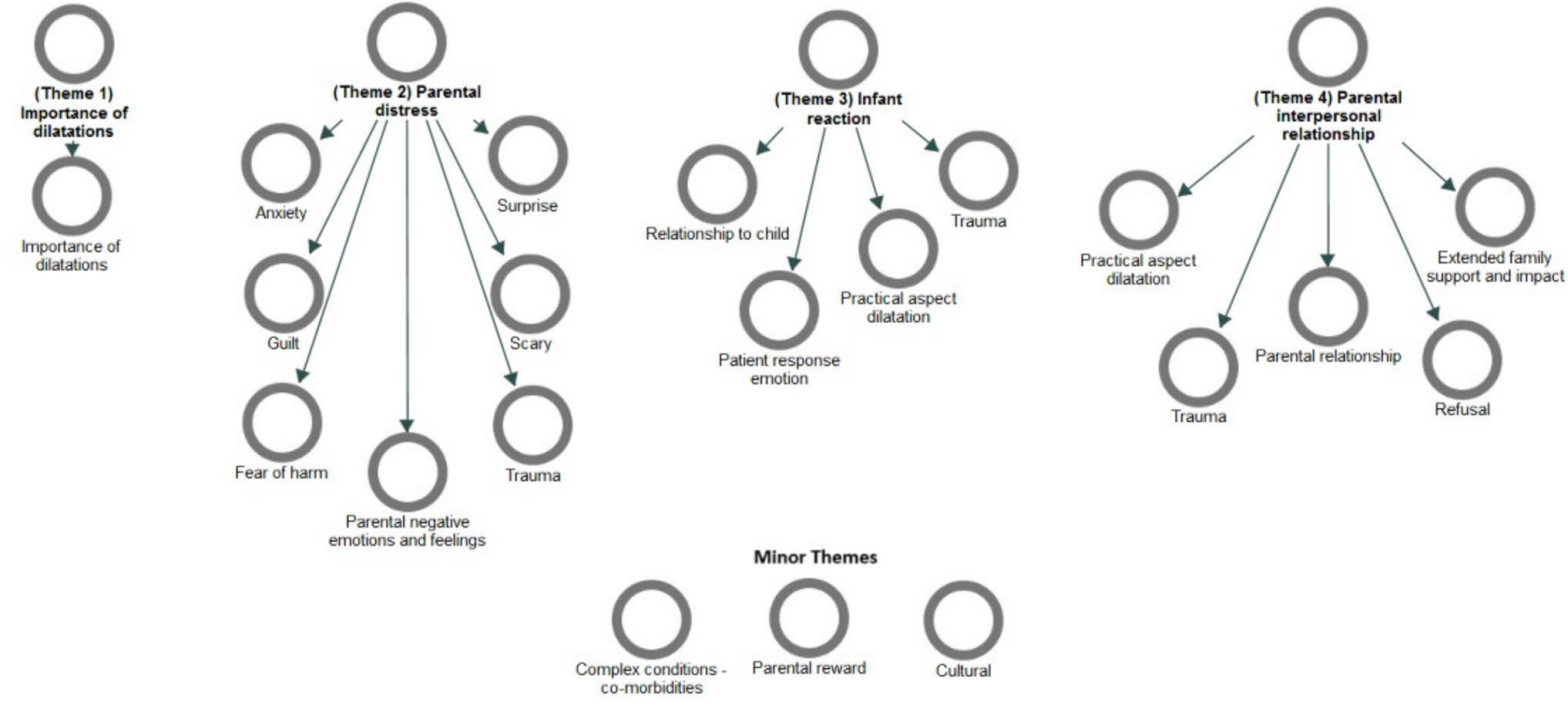
Thematic map and their contributory sub-themes and minor themes

There were four major themes identified, as summarised in Fig. 1 and Table 3. Additional minor themes were also identified and discussed further below.

**Table 3:** Summary of themes, sub-themes and illustrative quotes

Theme	Subtheme	Illustrative Quote
**Importance of dilatations** Parents submitted to medical expertise when evaluating the importance of dilatations, prioritising this over their experiences even when they had insight into wider potential social costs	–	“Well, if a doctor was told me to do so, they have the understanding and the knowledge and that’s what we will do.” **Participant 02** “What kind of parents are we if we let him allow him to become in a position where he needs a further corrective surgery just because we don’t like something?” **Participant 02** “We kind of had, you know, accepted her diagnosis and everything. Say we had to learn and do what is needed for her.” **Participant 04** “But for me in my head it was like this is my child and I need to do and as a mother I just feel like I will do whatever it takes. I never really focus too much on my partner and whether he’s there, whether it’s not. I just make sure that I was doing what I needed to do for my son.” **Participant 06**
**Parental distress, anxiety, guilt, fear of causing harm** A range of negative emotions were encountered by parents when performing dilatations which was in conflict to the sense of responsibility they felt	Anxiety	“Oh, I was just going. I just feel like my heart was beating fast and I don’t know how to deal with it.” **Participant 01** “felt frightened and worried” **Participant 08**
	Fear of harm	“And my partner was very reluctant to hold him because he found it very distressing, which then made the whole thing worse because X was wiggling. And then he was getting, you know, hurt.” **Focus group** “We don’t want to hold him too tight, but I was saying if you don’t hold him tighter, he is going to move in a way that this is going to seriously injure him. I need you to restrain him for him to be safe.” **Participant 02** “And as parents, obviously you don’t want to hurt your child” **Participant 12** “obviously not wanting to cause her any damage” **Participant 13**
	Guilt	“But essentially, he told me that if X then requires, if we didn’t do it, if I if I chose not to do it, that was his wording. If I chose not to do it and X later needed a second surgery to reopen that opening, it would be my fault.”** Participant 02** “We had a feeling we’re doing something not quite right that to him, you know what I mean?” **Participant 10** “One thing the way it affected me was like I have a screaming baby every day and I am causing her to scream” **Participant 05** “But I have hated a lot of stuff and this was a thing that I from the minute they said we had to do it. My heart has sank because I did not want to be the person to do that.” **Participant 02**
	Trauma	“It was a traumatic the whole thing was traumatic and I think like a lot of people have said, it’s not until afterwards you realise how traumatic it was.” **Focus group** “I was hurting him and I couldn’t explain him in a way that you would understand.” **Participant 03** “I thought I was even hurting more my son.” **Participant 03** “Whilst equally being in tears myself most days because I don’t like what I’m doing.” **Focus group** “Because a it wasn’t a very nice thing to do anyway for anybody. It’s not pleasant whether it goes well or it doesn’t, it’s still not something you ever wish to have to do.” **Focus group**
**Parents perceived a traumatic reaction from infants including anticipatory stress** Infants experienced pain, distress and anticipatory stress during dilatations which further compounded the range of negative emotions in their parents	Infant response	“Not very pleased by the whole thing.” **Focus group** “We still have them at home like the metal rods and I knocked them the other day when I was getting something out and he ran from the room” **Focus group** “He became more aware of what was going on in this period of time and he has become quite traumatised by a lot of different I mean every problem we go to now is a battle. It is tears everywhere. He is very reluctant to be touched by anyone but us that we’ve got lots of other issues going on.” **Focus group** “But during that period of doing the dilatations for the immediate half an hour after it didn’t want to come near me. And for me, that’s probably the biggest kicker of it. We’re fine now, but I know that he remembers it. I know that he, even if he doesn’t know what was going on, he’s still scared about that” **Focus group** “And he gets more like scared and shaking, and he’s like, crying.” **Participant 01** “He is very resistant. And ever since that operation, any time where we have had to take his nappy off in a hospital setting, we have had a meltdown of epic proportions and it got so bad I said in the first appointment that he would hear the packet of the metal rods being collected clinks. And even now he’s like there is a physical flinch reaction to it” **Participant 02** “What he sees the metal thing itself. He starts crying even before we touch him.” **Participant 10**
	Trauma	It was a traumatic the whole thing was traumatic and I think like a lot of people have said, it’s not until afterwards you realise how traumatic it was. **Focus group** “I thought I was even hurting more my son.” **Participant 03**
	Relationship to child	“And I don’t know for the future like how much does he actually remember? Will he remember how he felt about me during those moments? Because there was a definite didn’t want to touch Mummy after Mummy had done that to me for at least a little bit of time before he. Then, you know, all smiles and happy and cuddles before bed before the end of the night. But that was incredibly tough as a parent, not just how it impacted my relationship with my partner, but our family, but also with baby because well toddler by that point.” **Focus group** “Worse than I’d imagined, where he wouldn’t go to me afterwards because I was the one that was hurting him.” **Participant 02** “I read somewhere that somebody said if you think of it like you’re doing it for them, not to them, it made me. It was a better mindset to be in because I thought I’m doing it for him. It’s not. I’m not hurting him on purpose” **Participant 08**
	Practical aspect of performing dilatations	“It was a bit hard but I found my way around it because like you know, as mentioned earlier, it requires two people, but then my partner had to go to work and so you know, they need to be performed and you know, I had to find a way of, you know, manoeuvring my way around it.” **Focus group** “And we did struggle a little bit to start with” **Focus group** “But I think what about those parents that are single parents that have a parent that can’t? You know, I think people need to be trained how to do them by themselves.” **Focus group** “How I’m going to do this, but I know it needed to be done and then I just came up with this way of holding his legs somehow and doing it and thank goodness it worked out, but I think the training for you know, someone to do that, do it by themselves is important.” **Focus group** “If it has to be done, it has to be done. I just don’t think it should be done by parents.” **Participant 02**
**Detrimental impact on inter-parental relationships** A significant imbalance was identified amongst parents with fathers typically refusing to engage in dilatations. Parents also reported a strain developing in their relationship linking into the themes identified above	Paternal relationship	“and I feel that that had a huge impact on how we were able to administer it because it caused a lot of friction between me and my partner” **Focus group** “We had a very extended period of doing the dilatations and it did have a hugely negative impact on our relationship at the time.” **Focus group** “Argued about ever like full blown argument and that happened every single day for probably about six months.” **Focus group** “at home caused a lot of tension and stress.” **Focus group** “And we nearly broke up over it.” **Participant 02** “That is by far by far the worst thing that has impacted our relationship in the X’s nearly five years of life that if there was anything that was going to break us up, it was going to be that and it was at the point I was then the one that said I’m not doing this anymore because I was choosing my family over.” **Participant 02**
	Imbalance in roles	“And I’m mostly of the time I was alone with the care.” **Participant 03** “But we kind of got into a routine my husband couldn’t do. It wasn’t something that he felt he comfortable with doing.” **Focus group** “And my partner was very reluctant to hold him because he found it very distressing, which then made the whole thing worse because X was wiggling.” **Focus group** “We kind of, I mean, I did it, I did the dilatations. My husband did not want to do them.” **Focus group** “Y immediately said I can’t do it like he just point blank. I can’t. I can’t do it. And I was like, well, I guess that’s something that I’ll have to do then, as is most things that are for, like all the medical stuff.” **Participant 02**
***Minor themes*** **Complex co-morbidities** **Sense of reward** **Cultural implications**		“And then we learnt we had a chromosome condition as well, which kind of made things extra complicated” **Focus group** Now millions of people that we have seen very rarely do they consider X as a whole child rather than just their department, which makes it very difficult then to work out if the side effects we’re seeing. **Participant 02** “And I’m trying to encourage him to say, look, it’s going to be for the greater good at the end of this” **Focus group** “Yeah like I’m hoping it made a difference to her life.” **Participant 05** “Arabic society like background, if you know what I mean. So all of these sexual stuff is very restricted in our countries.” **Participant 10**

### Importance of dilatations

Parents unanimously reported their surgeons explained the importance of performing dilatations during their early consultations. Surgeons demonstrated the technique to families at different stages of the clinical journey. Parents were largely surprised with the responsibility of performing dilatations being bestowed upon them. Despite this, however, it was clear from both the focus group and interviews that they understood the potential significance of performing the procedure as presented to them. Compliance with the procedure was extremely high which emphasised the transference of belief in its importance with parents placing significant effect into successfully performing the dilatations and the direct impact it would have on outcomes for their child.

### Parental distress, anxiety, guilt, fear of causing harm

Whilst compliance with performing dilatations was universal, parents commonly expressed significant distress during the procedure that largely stemmed from what they perceived as a negative reaction from their child. The physical nature of the task that involved introducing a metal rod into the anus was perceived by the large majority of parents as an act that could potentially cause harm, triggering feelings of guilt. Caregivers also felt that they had to persist with the procedure as failure to comply would potentially detrimentally affect outcomes for their child adding a further layer of guilt to their emotions.

### Parents perceived a traumatic reaction from infants including anticipatory stress

The majority of parents reported perceiving stress and anxiety in their infant prior to and/or during performing dilatations. As a result of this, some parents felt that they were inflicting trauma on their child with a significant negative impact on their relationship with their child. This was particularly expressed by some mothers who felt that they had failed in their protective role as a parent. Parents felt that health professionals should ideally be the ones performing the dilatations so as to avoid having to place parents in the position of delivering potentially “traumatic” but felt to be necessary treatment.

### Detrimental impact on inter-parental relationships

The stress felt by caregivers in performing the dilatations also had an impact on their own relationship. Whilst some families were able to work through the dilatations together as a team, several others felt the entire experience for them as a unit was detrimental. Whilst most fathers were involved in performing dilatations, some completely refused to engage due to their perceptions of trauma and adverse feelings for themselves and their child. In the majority of cases, mothers, therefore, took the responsibility to lead and the imbalance in these roles was further contributing to the stress being experienced by families. In some cases, extended members of the family were able to lend support to parents, whereas others specifically chose not to disclose the need to perform dilatations to them.

### Additional minor themes

A few parents reported a sense of “reward” and feeling “proud” to have participated in their child’s reconstructive journey and achieving a positive end result. Only a minority, however, found the procedure straightforward and were confident in performing it without negative emotions.

Anorectal malformations are not always seen in clinical isolation. Parents of those infants with other associated anomalies found the management of multiple hospital appointments and procedures further compounded the stress of having to also perform dilatations at home. Information provision to parents on how to perform dilatations was largely to a very high standard with parents often receiving training directly from their lead surgeon.

One family also explained the internal battle they faced to comply with dilatations due to the act being against what they believed would be acceptable in their culture and community.

## Discussion

This study specifically looks to identify parental attitudes and experiences in performing dilatations through directly engaging with them in a focus group and semi-structured interviews. We were able to identify four discrete major themes that shed light on what has been previously been suspected but not established in evidence plus three minor themes worthy of further consideration in clinical practise. The importance of the procedure was universally understood by parents with each family receiving explicit instructions by their lead surgeon on its rationale and impact on future outcomes. However, we were able to also understand the impact of this on parents, infants and their relationships with each other. Our findings revealed the anxiety, trauma and guilt experiences by parents and significant anticipatory stress in their infants. Inter-parental relationships were also found to have been detrimentally impacted. Dilatations post-anorectal reconstruction are routinely recommended by paediatric colorectal surgeons worldwide. As part of Pena’s original description of the posterior sagittal anorectoplasty technique, daily dilatations were prescribed to prevent stricture formation [1]. The objective benefits of this were, however, challenged by Langer et al. [9] who identified no significant difference in stricture rates when infants underwent parental led daily dilatations versus surgeon-led weekly dilatations.

Randomised-controlled trials are considered gold standard in evidence-based medicine. The only such study till date exploring stricture rates has also reported no difference in objective outcomes [6]. Most recently, a systematic review assessing 7 studies with 400 patients also concluded that “universal” recommendation to perform dilatations should be questioned [11]. With the objective evidence to perform this procedure hence remaining equivocal, we sought to understand the parental view on performing dilatations on their infants.

Jenetzky et al. reported patient experience of dilatations in their cohort of patients. Pain was experienced in 69% of cases and bleeding in 32%. Similar experiences were reported by several parents in this study who described feelings of trauma and perceived harm being inflicted on their infants. The authors highlighted the lack of evidence to routinely recommend dilatations with their impact and need requiring further exploration [10]. In other published literature, the subjective experience of performing dilatations has previously been reported in specialist nursing staff but not directly on parental attitudes [12]. A qualitative approach was used in this study with one of the main themes identified as dilatations causing nursing distress. Furthermore, nurses had a universal impression that parents expressed a strong emotional reaction to dilatations [12]. This study was able to directly corroborate these impressions with parental distress, anxiety, guilt, fear of causing harm identified as one of our primary themes.

Our findings were in keeping with Diseth et al. who conducted a psychosocial study in 10 patients that had undergone surgery for low anorectal malformations [7]. The mean age of the participating adolescents in their study was 14.5 years with four patients remembering the experience of undergoing dilatations and two reporting hostility towards their mothers on account of pain. Parental experiences were also largely negative with mothers in particular finding the procedure difficult to perform due to evoking pain in their child. Several mothers also explained the “great protestations” they witnessed from their child and only one father helped in performing the dilatations which was very similar to our findings [7]. Mothers were thereby having to deal with an internal conflict of providing comfort and protection to their child whilst performing what they perceived was a traumatic procedure. Their stress was further compounded by the lack of support from fathers who largely refrained from engaging in dilatations. The same group of authors conducted a further study in 17 adolescents with 94% having undergone dilatations up to a median age of 2 years. Just over 50% of the parents in the study negatively described their emotions towards performing dilatations with 1/3 reporting a power struggle during the procedure. Parents also felt this procedure negatively impacted their relationship with their child and 59% felt it affected their inter-parental relationship and family life [8].

The impact of dilatations on future development has also been reported by the same authors above who identified the duration of dilatations as being the most important predictor of dissociative outcomes in adolescents and young adults [13]. The theories behind this are complex, however, can be partly explained with the switch in roles undertaken by the caregivers morphing into healthcare providers who inflict a “traumatic” procedure on their child not of out malevolence. This has also been reported in other cases of childhood trauma that has a detrimental impact on interpersonal relationships [13].

### Strengths and limitations

Our study population is limited to a single centre and, therefore, not necessarily representative of other cohorts, despite the resonance of our findings with published literature of observations in other patient cohorts [7, 8, 12]. Our findings provide a novel analysis of parental lived experience and learned expertise illuminating the importance of considering the wider social impact of clinical practise. Based on our experience of conducting the study we anticipate parental engagement in future co-designed care would be a useful approach to make improvements.

We limited our study cohort to those families whose children had undergone reconstructive surgery within 5 years of the study. This was performed to minimise recall bias. However, we also acknowledge that there are known long term effects of dilatations reported by adolescents and parents [8] which we did not capture through setting this exclusion criteria. We certainly feel this is worth exploring as an extension to a future study.

To mitigate for selection bias, the study investigators did not review patient outcomes prior to seeking consent for participation. However, we accept that non-response and self-selection biases could have impacted the composition of our study cohort.

SY’s role in the study as an “outsider co-investigator” to the field of practise brought a socioculturally informed critique to the design and delivery of the study. She worked with HT (as the senior surgical author and someone who does not routinely perform dilatations in his patient cohort) to ensure an open exploratory approach was taken.

## Conclusions

Parents who accept the importance of dilatations, are placing trust in their clinicians’ guidance despite their negative experiences of the intervention and perceived relational costs between themselves and their child. There is also evidence that dilatations performed in early life may have a detrimental impact on future psychosocial outcomes. As the clinical evidence for dilatation remains, at best, equivocal, we believe it should not be recommended as part of routine practise. Our research indicates that parental experiences are a vital aspect of care, and their perspectives should be considered before recommending routine dilatations. Further research engaging parents and older children in co-design of interventions to develop and communicate best practise, and decision-making in the current context of equivocal evidence is recommended.

## Data Availability

No data sets were generated or analysed during the current study.
